# Disseminated Cutaneous Mucormycosis Developing in Peristomal Pyoderma Gangrenosum

**DOI:** 10.7759/cureus.61137

**Published:** 2024-05-26

**Authors:** David A Prentice, Erasmia Christou, Wendy A Pearson

**Affiliations:** 1 Neurosciences, Perron Institute for Neurological and Translational Science, Perth, AUS; 2 Department of Internal Medicine, Royal Perth Hospital, Perth, AUS; 3 Department of General Surgery, Royal Perth Hospital, Perth, AUS; 4 School of Medicine, University of Western Australia, Nedlands, AUS; 5 Stomal Therapy Service, Royal Perth Hospital, Perth, AUS; 6 School of Nursing, Curtin University, Bentley, AUS

**Keywords:** biopsy, angioinvasive, lipase, fat necrosis, rhizopus, peristomal pyoderma gangrenosum, mucormycosis

## Abstract

A patient on long-term glucocorticoid therapy for peristomal pyoderma gangrenosum (PPG) who developed mucormycosis (MM) of the wound with dissemination was presented. The importance of skin biopsy, together with clinical evaluation in patients with PPG who are resistant to conventional therapy or who develop new symptoms related to their PPG is stressed. The risk and pathogenesis of invasive fungal infections with long-term corticosteroid therapy were explored. The epidemiology and detection of mucormycosis is discussed in this article.

## Introduction

Mucormycosis (MM)is a generic term for an invasive fungal infection by one of the species of Mucorales (i.e., *Rhizopus, Apophysomyces, Saksenaea, Cunninghaemella, Lichtheimia*). Overall, the most common infecting fungus is *Rhizopus oryzae*. Cutaneous MM is rare and often fatal if the diagnosis is delayed. The literature reports the mortality of adults with MM is significant. It ranges from 20% to 100% depending on patient comorbidities, the site of the infection, and variation in treatment [1]. There is evidence showing an increasing incidence of cutaneous MM due rising prevalence of diabetes and cancer immunosuppressive treatments. Most cases have been reported from the USA (37%) and Asia (31%), with Australia accounting for 5.8%. The anatomical sites involved are the lower extremities (26%), upper extremities (23%), abdomen (11%), and face (10%) [1]. 

Peristomal pyoderma gangrenosum is an uncommon peristomal skin complication and can be challenging to diagnose due to the lack of pathology and misdiagnosis [2]. Treatment often centres around topical and systemic therapies, especially the use of high-dose corticosteroids. The attendant metabolic and infective side effects of which are well known. Peristomal pyoderma gangrenosum, if diagnosed correctly using the proposed criteria by Su et al. [3] or Maverakis et al. [4], can be responsive to high-dose corticosteroids but healing may be prolonged, and recurrences are recognised. To reduce this risk, the authors published a series of patients who showed a remarkable response to a crushed prednisolone regime combined with hydrocolloid powder [2]. We report a patient who developed an invasive MM (*Rhizopus oryzae*) in the PPG wound with fatal consequences. The immunosuppressive effect of high-dose corticosteroids as a result of the mainstay treatment of PPG may increase the risk of developing MM in the PPG wound, and therefore, an increased awareness of opportunistic infections is required.

## Case presentation

A female in her 70s was in a rehabilitation facility for functional decline. The patient had undergone a radical cystectomy with the formation of an ileal conduit 10 years previously for muscle-invasive bladder cancer. Significant past medical history included, left nephrectomy, chronic kidney disease, bronchiectasis, type two diabetes, atrial fibrillation, ischemic heart disease, breast cancer (treated with bilateral mastectomy, chemotherapy, and radiotherapy), cholecystitis managed with a cholecystostomy tube and a persistently elevated serum lipase without any ongoing evidence of pancreatitis.

**Figure 1:**
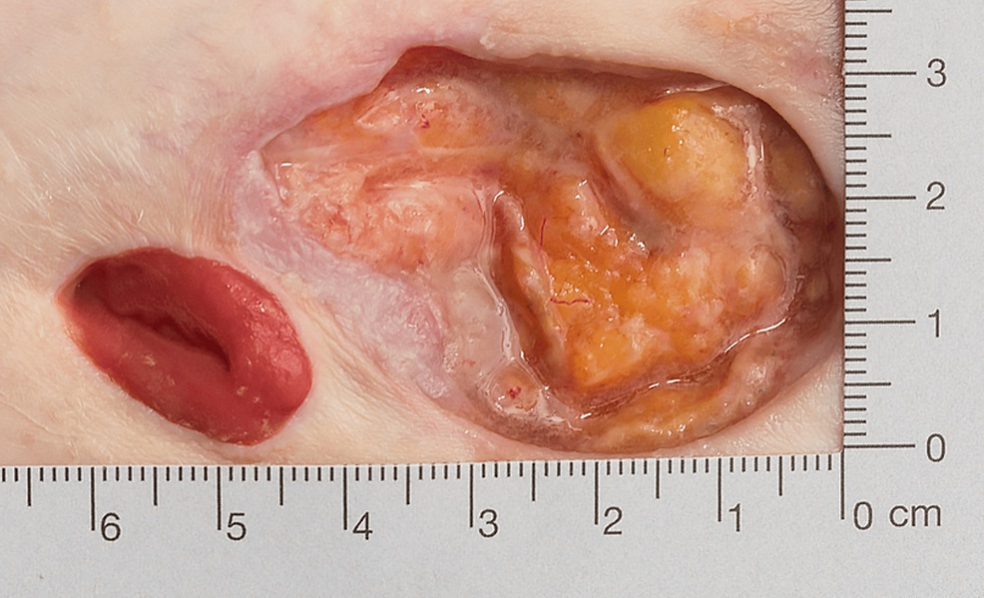
Healthy ileal conduit with healing peristomal pyoderma gangrenosum

Peristomal pyoderma gangrenosum was diagnosed both clinically and on subsequent biopsy nine months prior to presenting with MM. The PPG was initially treated with the crushed prednisolone/hydrocolloid powder regime, as discussed above [2], with limited response. After several months of the topical therapy, our patient was subsequently commenced on high-dose oral prednisolone (50 mg daily) in conjunction with the continuation of the topical crushed prednisolone/hydrocolloid powder therapy with a corresponding reduction in the PPG but not complete healing (Figure 1). Gradual weaning of the oral prednisolone commenced and managed to reduce to 15 mg daily due to glucocorticoid myopathy and worsening of diabetic control. Three days prior to her transfer to a tertiary hospital, she had central abdominal pain, constipation, and nausea. This coincided with the worsening appearance of her peristomal abdominal wound, with the area of necrosis surrounding her ileal conduit. Prior to transferring our patient to the tertiary centre, she was still on oral prednisolone and was commenced on augmentin, vancomycin, and fluconazole.

On the day of transfer from the peripheral rehabilitation unit to a major tertiary hospital in Western Australia, it was noted that the PPG wound was erythematous but not grossly infected. Haemoglobin was 10.2 g/dl, C-reactive protein was elevated at 98 mg/l, creatinine was 205 mg/dl, and her white cell count had been elevated in the days leading up to transfer but had stabilised. There was sloughy devitalised material in base of wound and dermatological recommendation was that of continued management with glucocorticoids, antibiotics, and wound dressings. Surgical debridement was decided against in the first instance as operative management can be known to worsen PPG [5].

**Figure 2:**
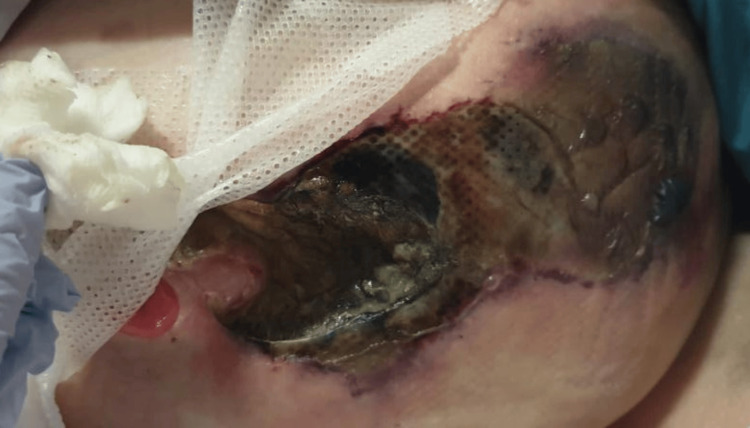
Appearance of the peristomal wound prior to surgical debridement

**Figure 3:**
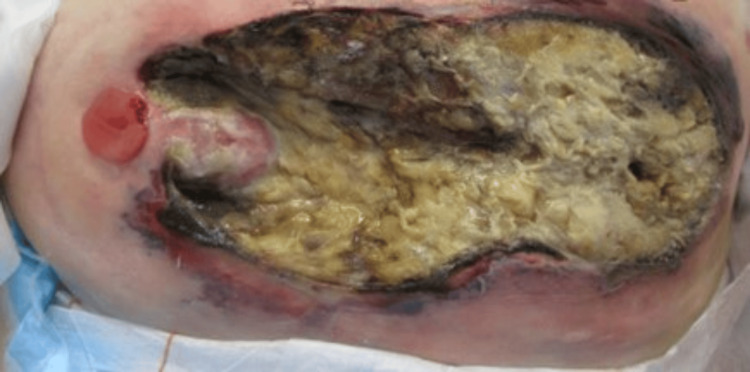
Peristomal wound three days after surgical debridement

The patient had ongoing clinical decline over the next three days with worsening sepsis, increasing drowsiness, and deterioration in the appearance of the peristomal wound and surrounding area (Figure 2). After re-discussion with all specialty teams, it was agreed she would need wound debridement under local anaesthetic with sedation for symptomatic control only. At this point in time, our patient was a poor surgical candidate, and although for treatment, the goals of care were not for intubation, intensive care, or resuscitation. During the procedure, there was found to be 15 cm of necrotic skin and subcutaneous fat on the lower abdominal wall surrounding the ileal conduit. The ileal conduit itself was healthy, intact, and still draining urine. Slough overlay deep fascia but there was no evidence of necrotising fasciitis (Figure 3).

**Figure 4:**
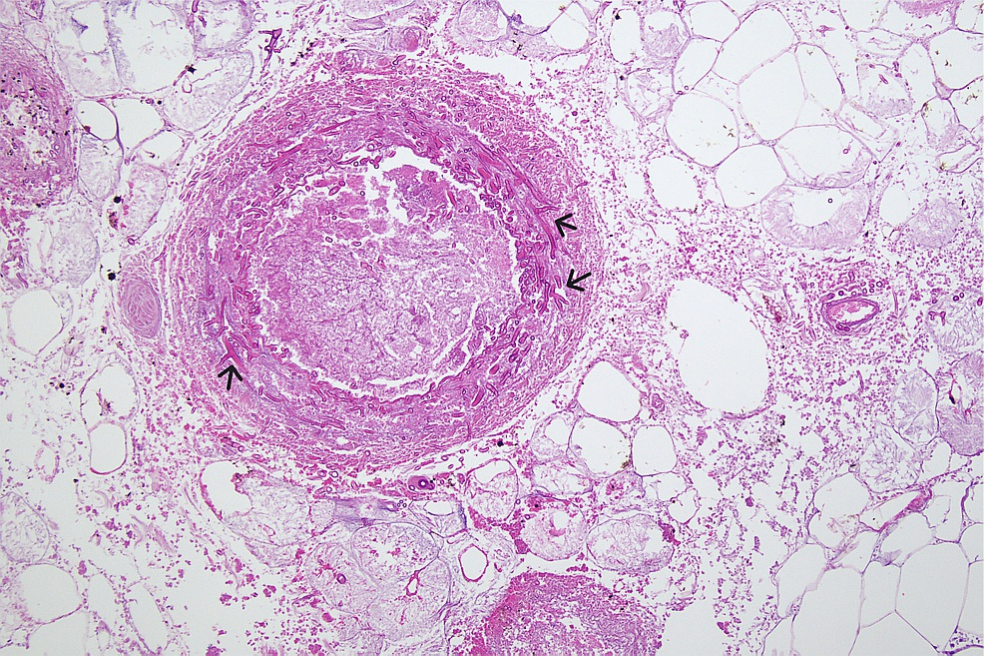
Histopathology images High power hematoxylin and eosin stained section showing vascular invasion and thrombosis with fungal hyphae which are broad and non-septate consistent with mucormycosis.

Postoperatively, the patient experienced ongoing signs of sepsis with hypotension and the development of spontaneous bruising and satellite lesions consistent with MM dissemination. These lesions were not biopsied, and blood cultures were negative for fungi. Histopathology confirmed angio-invasive mucormycosis with adjacent widespread fat necrosis and epidermal lysis with congestion and coagulation (Figure 4). Fungal organisms were found extending down to the margin of deep fascia and fungal DNA was matched for *Rhizopus oryzae*. Fluconazole and vancomycin were subsequently ceased, augmentin was continued, and liposomal amphotericin B was added.

The patient was also noted to have a widespread purpuric rash that developed over the last couple of months, with worsening appearances during this admission, including ecchymosis and bullae. Overall, the patient had poor physiological reserve and was not a suitable candidate for further radical debridement. Given the aggressive nature of the disease and the associated poor prognosis, she was subsequently palliated and died in hospital 10 days after her transfer.

## Discussion

Our case illustrates the importance of considering other pathologies in patients with PPG who fail to heal or who develop new symptoms. Skin biopsy is often avoided in PPG for fear of worsening the wound due to the pathergy response. A pathergy response occurs in at least 30% of PG patients [6]. This is only the fourth published and third fatal case of MM developing in a PPG wound. All the previous published cases had a faecal stoma [7-9]. The widespread purpuric rash that evolved and worsened during this admission, including ecchymosis and bullae, was initially thought to be medication-related or coagulative skin changes secondary to sepsis, but subsequent review of the patient could be disseminated secondary MM.

General mucormycosis

Mucormycosis is classified into types based on its location. It is caused by order of Mucorales which include the Genera of Rhizopus. Rhizopus species, especially *R. oryzae,* account for 70% of cases [10]. The clinical syndromes include rhinocerebral, cutaneous, pulmonary, and gastrointestinal (stomach, colon) MM. Mucormycosis is ubiquitous and found in decaying plants, soil, and foods [1]. 

The risk for invasive MM is immunosuppression from haematological malignancies, solid organ transplantation, trauma, burns, diabetes, prolonged glucocorticoid usage, hemochromatosis, deferoxamine use, and intravenous drug usage [11]. It is well-recognised that immunocompetent patients, especially those with trauma, can also be infected. The recent outbreak of rhino cerebral and pulmonary MM in coronavirus 2019 (COVID-19) Indian patients has variously been attributed to diabetes incidence, dexamethasone, and zinc therapies [12].

Cutaneous mucormycosis

Mucormycosis cannot penetrate intact skin. Disruption of the integumentary system is a prerequisite for infection. Trauma (car accidents, tornados, tsunamis) and burns patients constitute many of the reported cases [13]. Cases in immunocompetent patients have even occurred from intravenous lines and insect bites [13]. The classical appearance of cutaneous MM is black eschar, but it can be pustular, ulcerative haemorrhagic, or even vesicular [13]. Cutaneous MM can also present with a “bull's-eye“ lesion, with either the centre being pale necrotic or haemorrhagic with a red halo [13]. Cutaneous dissemination leads to satellite haemorrhagic lesions, often presaging a fatal outcome [13].

Diagnosis

Diagnosis can be difficult and requires a high index of clinical suspicion. Fungal cultures or the lesions are rarely diagnostic (< 50%) and a tissue biopsy showing angioinvasive disease is required to make a firm diagnosis. In disseminated disease, blood cultures are rarely positive, demonstrated by only three case reports in the literature [14]. 

Molecular techniques are used to positively diagnose MM using a semi-nested PCR targeting the 18S RNA gene of Mucorales. Multiplex real-time quantitative PCR (qPCR) targeting ITS1/ITS2 region or amplifying the cytochrome b gene can identify subspecies (Rhizopus and Mucor) The same technique has been used on blood samples with a sensitivity of 81-92% [15]. Involvement in the subcutaneous fat is common with extensive fat necrosis as was presented by our patient. We speculate the persistent elevation of our patient’s serum lipase might be due cross to a reaction of the Rhizopus lipase with human serum lipase.

Glucocorticoids and fungal risk

Long-term use of glucocorticoids increases the risk of MM and carries an increased risk (four-fold) of death from the MM infection [16]. The major immune response to fungal infection is via the innate defence system with phagocytosis by neutrophils and macrophages. Fungal cell walls are recognised through direct pattern recognition or via opsonisation with complement. Corticosteroids have multiple effects in increasing the MM risk by limiting vasodilation, preventing the egress of neutrophils at the infection site, inhibiting bone marrow production, releasing neutrophils and monocytes, and suppressing phagolysosome membrane fusion in macrophages. They also inhibit fungal killing by reduction of oxidative products in the lysosome [16]. In vitro studies of glucocorticoids (dexamethasone and hydrocortisone) have been shown to increase the growth of Aspergillus, but this has not been shown with Rhizopus [17].

Prevention

Prevention of the risk of MM infection is by reduction of dose and duration of corticosteroids or cessation of them if it is safe. Acetic acid in low concentrations has been shown to kill Mucorales species in vitro [18]. This could be applied locally if colonisation is detected. Statins are also an antifungal in serum concentrations achieved with standard oral therapy [17]. None of the three patients reported in the literature were documented to be taking these medications [7-9]. Interestingly, prolonged glucocorticoid therapy was the major risk factor and accounted for 37% of MM infections [1]. 

Hospital devices

Mucormycosis has been associated with hospital devices [19]. There was an outbreak of cutaneous MM with the use of ostomy pouches containing karaya as the baseplate [19], but karaya bases have long since been replaced with hydrocolloids, as was the case with our patient. Wooden tongue depressors, adhesive tapes, bandages, insulin needles, catheters, and drains have all been implicated in MM infections [20]. We could not implicate any of these devices as the causative agent in our patient.

## Conclusions

In this article, we describe the fourth case of MM arising in PPG in the literature and the first involving a urinary stoma. Cutaneous MM is a recognised complication of severe trauma, organ transplantation and haematological malignancies but is also associated with long term glucocorticoid use. The cutaneous features can be characteristic with central necrosis with surrounding erythema and satellite lesions but can also be non-specific. A higher degree of suspicion of MM is required with a deep tissue biopsy together with histological and molecular markers for MM. There is a significant mortality rate if not diagnosed early and requires debridement and appropriate antifungal therapy to be urgently commenced.

Peristomal pyoderma gangrenosum is rare and can be difficult to diagnose because of confusion with other more common peristomal ulcerative aetiologies. The current mainstay of treating PPG is with high dose oral corticosteroid resulting in significant immunosuppression if continued for a length of time. Peristomal pyoderma gangrenosum that fails to heal with either oral and/or topical immunosuppressive therapy should be re-evaluated with wound swabs and tissue biopsy, despite the risk of pathergy, especially if the patient is on long term corticosteroid therapy. In addition, a multi-disciplinary team approach with an experienced dermatologist, infectious disease specialist, the treating medical team and stomal therapy nurse should be obtained.
